# pCLIF-SOFA is a reliable outcome prognostication score of critically ill children with cirrhosis: an ESPNIC multicentre study

**DOI:** 10.1186/s13613-020-00753-w

**Published:** 2020-10-14

**Authors:** Caroline Claude, Akash Deep, Martin Kneyber, Salman Siddiqui, Sylvain Renolleau, Luc Morin, Emmanuel Jacquemin, Jean-Paul Teglas, Vincent Gajdos, Pierre Tissières, Philippe Durand, the ESPNIC liver failure, support working group

**Affiliations:** 1 Paediatric Intensive Care and Neonatal Medicine, AP-HP Paris Saclay University, Bicêtre Hospital, 94275 Le Kremlin-Bicêtre, France; 2 Paediatric Intensive Care Unit, King’s College Hospital NHS Foundation Trust, London, UK; 3 Department of Paediatrics, Division of Paediatric Critical Care Medicine, Beatrix Children’s Hospital, University Medical Center Groningen, University of Groningen, Groningen, Netherlands; 4 Paediatric Intensive Care, Necker Hospital, AP-HP, Paris, France; 5 Paediatric Hepatology, AP-HP Paris Saclay University, Bicêtre Hospital, Le Kremlin-Bicêtre, France; 6 INSERM 1018, AP-HP Paris Saclay University, Bicêtre Hospital, Le Kremlin-Bicêtre, France; 7 Institute of Integrative Biology of the Cell, CNRS, CEA, Paris Saclay University, Gif-sur-Yvette, France; 8 European Society of Paediatric and Neonatal Intensive Care, Geneva, Switzerland

**Keywords:** Cirrhosis, ACLF, Sepsis, Bilirubin, INR, pCLIF-SOFA, Predictive factors, Liver transplantation

## Abstract

**Background and aims:**

Data on outcome of critically ill children with cirrhosis are scarce. We aimed to evaluate the prognostic accuracy of sequential organs scoring systems in children with cirrhosis admitted to Paediatric Intensive Care Units (PICU).

**Methods:**

We performed a multicentre retrospective analysis of children with cirrhosis admitted into four European PICUs between 2011 and 2016. Investigators were members of the ESPNIC liver failure and support working group. Paediatric End-Stage Liver Disease (PELD) and paediatric chronic liver failure sequential organ failure assessment score (pCLIF-SOFA) diagnostic accuracy for 28- and 60-day liver transplantation, 28-day mortality and 60-day composite outcome (ie. death or liver transplantation) were tested.

**Results:**

One-hundred-and-thirty children were included. The main causes for PICU admission were acute-on-chronic liver failure (ACLF), gastrointestinal bleeding and sepsis. Twenty-nine percent died and 22.3% were transplanted by day-60 after PICU admission. On multivariable analysis, pCLIF-SOFA was the only predictor of mortality at day-28 and of composite outcome. Both pCLIF-SOFA and ACLF were independently associated with emergent liver transplantation. The pCLIF-SOFA score higher than 9 well predicted a 28-day mortality with a sensitivity of 87.8% and a specificity of 77.3%. A pCLIF-SOFA score higher than 7 was independently associated with liver transplantation on day-60. Stage 3 AKI assessed with KDIGO classification was significantly associated with 28-day mortality.

**Conclusions:**

Half of critically ill cirrhotic children admitted to PICU either died or were transplanted within the initial 28-day period. On admission pCLIF-SOFA score accurately identify patients transplanted at day-28 and day-60 to those alive without LT and is associated with 28-day mortality and composite outcome at day-60.

## Introduction

Cirrhosis still remains a life-threatening condition in the era of liver transplantation and several complications involving organ failures, gastrointestinal bleeding, and septic shock may impair outcome [1]. In adults, many clinical studies report a poor prognosis in critically ill cirrhotic adult patients requiring ICU admission [2]. Paediatric liver diseases have seen their prognosis challenged due to the progress of liver transplantation (LT) [3]. In Europe, the indications for paediatric LT are mainly chronic liver diseases, with metabolic and cholestatic diseases encompassing the majority of causes. Although there is paediatric data on global and long-term prognostication of various cause of liver disease, there is little data specifically on cirrhosis in children admitted to Paediatric Intensive Care Unit (PICU) while awaiting LT. Delay on LT list enrolment has shown to be correlated with outcome in infants [4]. Pre-transplantation complications arise mainly at the cirrhosis stage. Death of children with acute decompensated cirrhosis has been reported to be of 33% and mortality increases with the number of failing organs [5, 6]. A better understanding of the clinical pathway and unfavourable outcome in cirrhotic children, especially when admission to PICU is needed, could improve medical management as well as organ allocation prioritization in these children. In the present study, we aim to describe the characteristics of cirrhotic children admitted in four European PICUs and assess sequential organ failure scores in regard to mortality, liver transplantation at days 28 and 60.

## Materials and methods

### Patients and outcomes

We performed a retrospective multicentre study of all children less than 18 years old with non-transplanted cirrhosis admitted to PICU between January 2011 and January 2016 in four paediatric liver transplantation centres (France, United Kingdom, Netherlands). Patients were identified using institutional electronic database. The diagnosis of cirrhosis was based on previous liver biopsy findings or a composite of clinical, laboratory and imagery data in accordance with published guidelines at the discretion of staff hepatologist [7]. The project was approved by the French Intensive Care Ethic Committee (CE SRLF 19–21) and was granted a waiver of informed consent. The primary endpoint was, in survivors at day-28, LT at day-28. The three secondary outcomes studied were (1) in survivors at day-60, LT at day-60; (2) mortality at day-28; (3) a combined criterion including LT or death at day-60. In addition, role of acute-on-chronic liver failure (ACLF), sepsis, centre and presence of comorbidities will be assessed. Day-28 and day-60 included PICU or Hospital data.

### Data collection

We collected standard data on history, main indications for PICU admission, physical examination, laboratory measurements at admission and day-7, and adverse events until discharge from the PICU and from the hospital. All collected data were available in all four centres. Aetiologies were classified as metabolic, infectious, drug-related, auto-immune or cholestatic diseases. Complications due to cirrhosis pre-existing to PICU admission were reported: growth failure, osteopenia, portal hypertension including variceal-bleeding history, spontaneous bacterial peritonitis (SBP), hepato-pulmonary syndrome, hepatopulmonary syndrome (HPS), porto-pulmonary hypertension, or hepatocellular carcinoma. Reason for admissions included ACLF, gastrointestinal (GI) bleeding, sepsis, cardio-respiratory failure, acute kidney injury, neurologic failure. The adverse events during PICU stay were recorded if they were different from the admission indication. These included acute kidney injury (AKI), sepsis, hepatic encephalopathy (HE) and its grading according to the Trey classification, bleeding, metabolic disturbances (hypoglycaemia, hyperammonemia), death or LT. The variables collected were used to compute one score of severity at admission (PIM2: Paediatric Index of Mortality 2), two scores of organ failure during the first 24 h: PELD, pCLIF-SOFA [5, 8, 9] (Additional file 1), and worst KDIGO (Kidney Disease Improving Global Outcomes) classification during hospitalization [10].

### Definitions

Acute-on-chronic liver failure (ACLF) was defined as an impairment of hepatic functions in cirrhotic patients, due to a triggering factor and resulting in at least two organ failures (European Association for the Study of Liver-Chronic Liver Failure, EASL-CLIF definition) [11]. AKI was defined according to KDIGO definition into three stages [10]. Sepsis was defined following the Sepsis-3 definition with a paediatric adaptation of organ failures criterion according to Goldstein, as an infection with two or more organ failures [12, 13]. On admission, patients with ACLF triggered by sepsis were classified as ACLF. Pathogen and sites of infection (pneumonia, urinary tract infection, SBP, cholangitis, primary bloodstream infection, meningitis or catheter-related bloodstream infection) were reported. GI tract bleeding, neurological impairment (cerebral oedema, HE, or seizures), acute respiratory and hemodynamic failure were also considered.

### Statistical analysis

The demographic and clinical characteristics of patients were assessed for normality using Kolmogorov–Smirnov test and expressed as numbers and percentages for binary or ordinary data and median and interquartile range (IQR) for continuous data. To evaluate predictors of outcome, in addition to calculated scores, clinically pertinent criterion from admission time (total bilirubin, INR, presence of comorbidities, centre) and during the stay (sepsis, ACLF) were selected. For multivariable analysis, pCLIF-SOFA, ACLF, sepsis, centre and presence of comorbidities were included. The *t*-test was used to compare continuous variables between two different groups and Chi-square was used to compare categorical variables (or exact Fisher test when expected values are less than 5). The prognostic performances of the both PELD and pCLIF-SOFA score were compared by drawing a receiver operating characteristic curve and the area under the curve (AUC) calculated through logistic linear predictors. The best thresholds were obtained with the calculation of sensitivity, specificity, positive and negative predictive values and the Youden’s index (sensitivity + specificity-1). We used logistic regression to estimate odd ratios (OR) and 95% confidence intervals (95% CI). Logistic regression multivariable analysis using a descending incremental (or a backward elimination) method with a stopping threshold of 0.2 was used to identify independent predictors for each outcome. For all analysis, survival without LT was considered as reference. Survival of patients according to KDIGO score have been evaluated by Kaplan–Meier curves and these later have been tested using log-rank test. Centres effect on mortality has been tested using Cox regression. All analyses were conducted using Stata software, version 14 (StataCorp). *p* < 0.05 was considered statistically significant.

## Results

### Study population characteristics

A total of 130 patients were enrolled with a median age of 41 [1–200] months. The majority (*n* = 120, 92.2%) had either a metabolic or a cholestatic (including biliary atresia) cirrhosis. The main reason requiring PICU admission was GI tract bleeding (42/130, 32.3%) followed by ACLF (23/130, 17.7%) and sepsis (24/130, 18%). In addition, 6/23 patients with ACLF had a concomitant sepsis at admission. Patient’s clinical and biological characteristics on admission are shown in Table 1.

**Table 1:** Characteristics and severity parameters of patients at admission

Characteristics	Value^a^
Centres	
Centre 1	38 (29.2%)
Centre 2	33 (25.5%)
Centre 3	31 (23.8%)
Centre 4	28 (21.5%)
Cause of cirrhosis	
Metabolic	28 (21.5%)
Cholestatic including BA	92 (70.7%)
Auto-immune	4 (3%)
Drug	4 (3%)
Infectious	2 (1.5%)
Female	58 (44.6%)
Age (months)	41 [1.0; 200]
Weight (kg)	8.7 [6.0; 18.5]
Reason for admission	
ACLF	23 (17.7%)
GI bleeding	42 (32.3%)
Neurological	4 (3%)
Respiratory	18 (13.8%)
Sepsis	24 (18.4%)
Other	19 (14.6%)
Bilirubin (µmol/L)	216 [89; 403]
INR	1.89 [1.2; 3.42]
pCLIF-SOFA score	8.6 [0; 21]
PELD score	18.6 [– 14.7; 47.9]
KDIGO stage (1 + 2, 3)	17 (13%), 23 (17.7%)
PIM2 score	13.4% [12;4; 14.4]
Mechanical ventilation	35 (27%)
Sepsis and septic shock (admission + hospitalization)	30 + 34 (49.2%)
Sepsis	38 (59.4%)
Septic shock	26 (40.6%)
PICU-LOS (days)	13 [2; 17]

*BA* biliary atresia, *ACLF* acute-on-chronic liver failure, *GI* gastro-intestinal, *INR* International Normalized Ratio, *pCLIF-SOFA* Paediatric Chronic Liver Failure-Sequential Organ Failure Assessment score, *PELD* paediatric end-stage liver disease, *KDIGO* Kidney Disease Improving Global Outcomes, *PIM2* Paediatric Index of Mortality 2, *PICU-LOS* paediatric intensive care unit-length of stay

^a^Median [IQR] or number (percentage) as appropriate

**Fig. 1:**
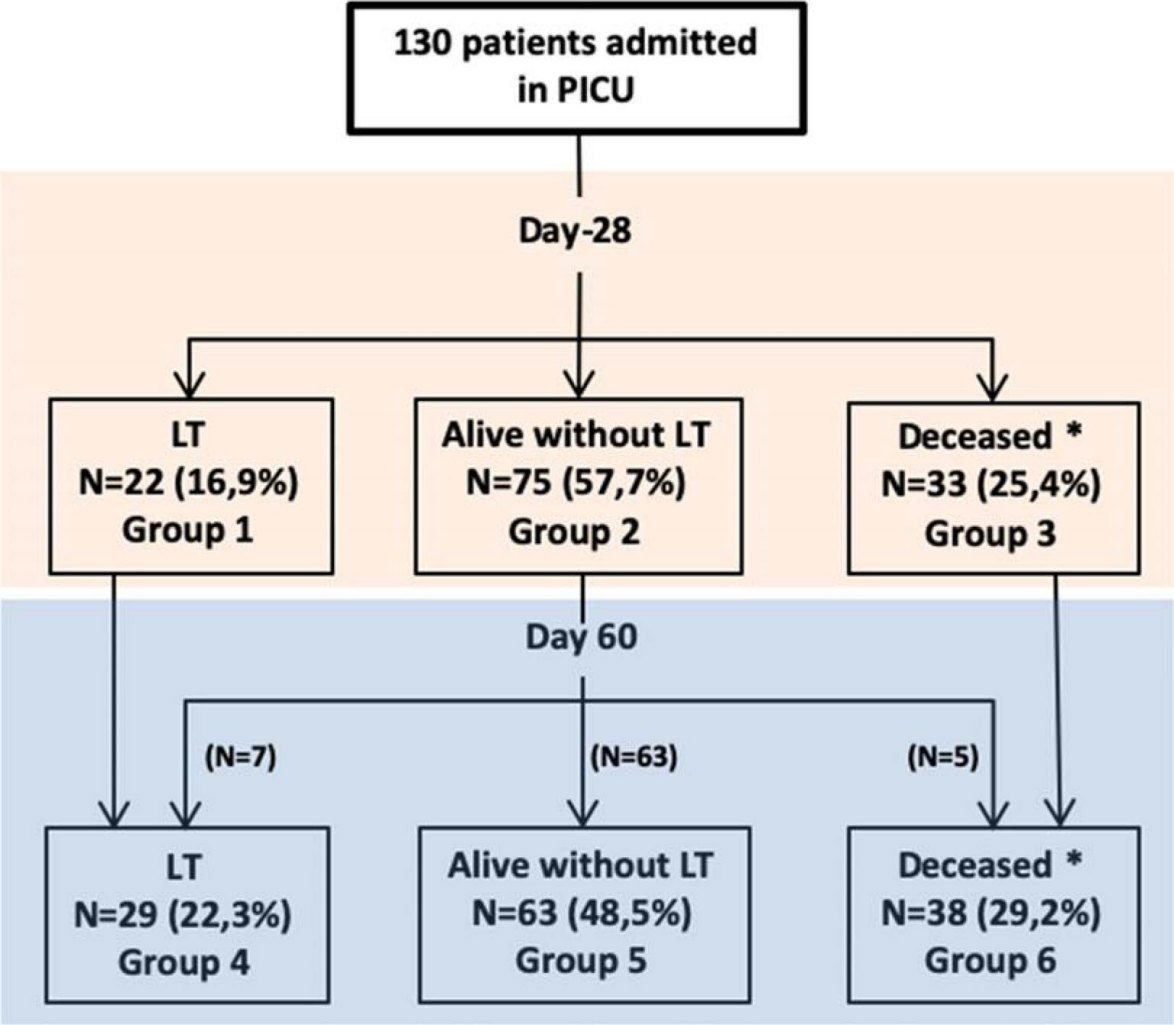
Flowchart of the 130 children with acute decompensated chronic liver disease admitted in PICU and outcomes analysis. *No death in liver transplanted patients. Outcome analysis: primary outcome: group 1 versus group 2; secondary outcome 1: group 4 versus group 5; secondary outcome 2: group 3 versus group 2; secondary outcome 3: groups 4 + 6 versus group 5. LT, liver transplantation

The median PICU stay was 13 [2–17] days. At day-28, 33/130 (25.4%) patients died, and 22/130 (16.9%) received an emergent LT. At day-60, a total of 29/130 patients (22.3%) were transplanted, of those none died, and 63/130 (48.5%) patients survived without LT (Fig. 1). During the whole PICU hospitalization, sepsis (including 30 from admission) occurred in 64/130 (49.2%), 26/64 (40.6%) of those were in septic shock. Although identified in univariate model, sepsis was not associated with primary and secondary outcomes on multivariable analysis. Bacterial infections accounted for the majority of sepsis and Gram-negative bacteria were the most common yielded pathogens (41% identified). The main site of infection was SBP (27%) followed by catheter-related bloodstream infection (24%). Most children (103/130, 79.2%) required mechanical ventilation during their stay, 35/103 from admission and 68/103 during their stay, respectively. Of these 103 ventilated patients, 33 (32%) died on the 60th day. Two out of eight patients were known to have hepato-pulmonary syndrome on admission, died. Regarding renal failure, 23/130 (17.7%) developed stage 3 AKI and 15/130 (11.5%) patients were dialysed during PICU stay of whom 7/15 (46.7%) died. The Kaplan–Meier survival curve at day-28 showed a significant survival difference between the three groups: no AKI, stage 1 + 2, and stage 3 (log-rank *p* = *0.0001*; Additional file 2). No centre effect on day-28 and day-60 mortality was found (respectively, *p* = 0.18 and *p* = 0.12; Cox regression analysis).

### Predictors of outcome

**Fig. 2:**
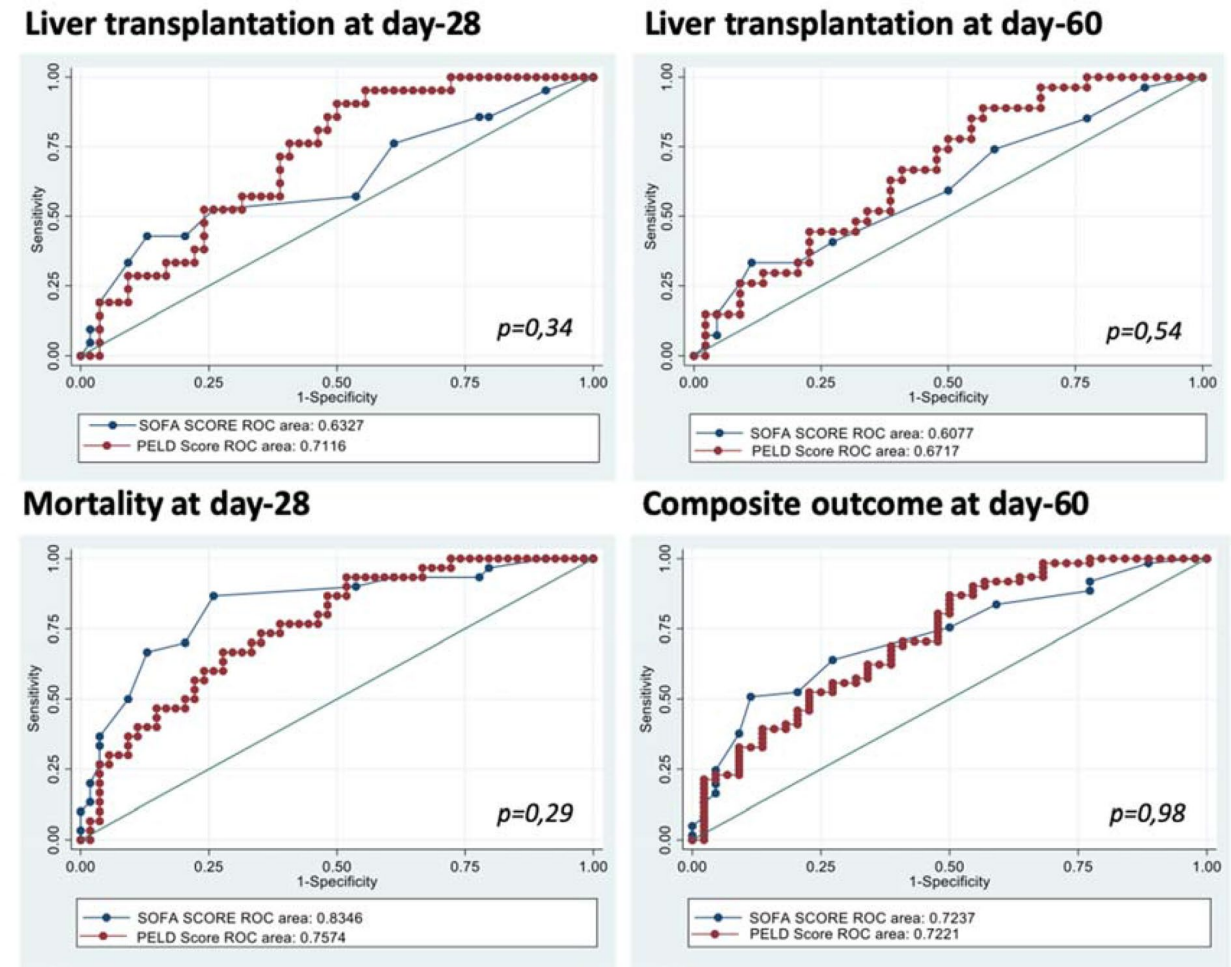
Receiver operating characteristics curves showing the discrimination ability of the pCLIF-SOFA and PELD scores in predicting liver transplantation on day-28, day-60, 28-day mortality, and 60-day composite outcome. All comparisons were against survival without liver transplantation

Out of the three tested scores, only PIM2 failed to show a significant difference in all primary and secondary outcomes (Additional file 3). Unsurprisingly, both pCLIF-SOFA and PELD showed a similar diagnostic performance with all outcomes (Fig. 2). This is closely related to the fact that the two PELD criteria (total bilirubin and INR) with the higher impact on scoring are included in the pCLIF-SOFA score. Considering that pCLIF-SOFA score better describe conditional organ failures which is of specific relevance for acutely ill cirrhotic patients admitted in PICU, we further tested only the pCLIF-SOFA. On multivariable analysis, ACLF and pCLIF-SOFA were both independently associated with the primary outcome (Table 2). On day-28 and day-60, a pCLIF-SOFA ≥ 7 had a sensibility and specificity for liver transplantation of 77.3%¨/44% and 75.9%/47.6%, respectively. Performance of pCLIF-SOFA for identifying patients with day-28 mortality was high [AUC ROC 0.83 (95% CI 0.74–0.93), *p* < 0.001]. Best cut-off value for day-28 mortality prediction was 9 with a sensitivity of 87.8% and specificity of 77.3%. On multivariable analysis, pCLIF-SOFA ≥ 7, ACLF and presence of comorbidities were associated with emergent LT at day-60 (Table 3), whereas only pCLIF-SOFA remained associated with mortality at day-28 (Table 4) and composite outcome at day-60 (Table 5). No centre effect was found for primary outcome (LT day-28, *p* = 0.34; LT day-60, *p* = *0.13*) nor for the composite outcome (*p* = *0.44*).

**Table 2:** Risk factors for liver transplantation (*n* = 22) versus survival without (*n* = 75) on day-28

Characteristics	Univariate analysis		Multivariable analysis	
	Odds ratio	*p* value	Odds ratio	*p* value
Comorbidity	0.18 (0.06–0.56)	0.003	0.03 (0.00–0.53)	0.017
Growth failure	0.80 (0.26–2.48)	0.711		
ACLF	17.75 (4.79–65.7)	0.000	6.12 (1.06–35.03)	0.042
Sepsis	1.58 (0.60–4.12)	0.344		
Biliary atresia	1.79 (0.67–4.12)	0.241		
INR ≥ 2.5	5.22 (1.88–14.5)	0.001		
Bilirubin ≥ 300 μmol/L	2.88 (1.00–8.30)	0.050		
pCLIF-SOFA	1.21 (1.04–1.40)	0.011	1.3 (1.04–1.67)	0.019
PELD	1.06 (1.01–1.11)	0.009		

Odds ratio are expressed as 95% (confidence interval). On multivariable analysis, centre effect was not significant (*p* = 0.20)

*ACLF* acute-on-chronic liver failure, *BA* biliary atresia, *INR* International Normalized Ratio, *pCLIF-SOFA* Paediatric Chronic Liver Failure-Sequential Organ Failure Assessment score, *PELD* paediatric end-stage liver disease

**Table 3:** Risk factors for liver transplantation (*n* = 29) versus survival without (*n* = 63) on day-60

Characteristics	Univariate analysis		Multivariable analysis	
	Odds ratio	*p* value	Odds ratio	*p* value
Comorbidity	0.12 (0.04–0.35)	0.001	0.10 (0.03–0.33)	0.001
Growth failure	2.02 (0.76–5.39)	0.158		
ACLF	9.01 (2.55–31.78)	0.001	5.02 (1.24–20.23)	0.023
Sepsis	1.74(0.71–4.25)	0.225		
Biliary atresia	1.37 (0.56–3.34)	0.485		
INR ≥ 2.5	2.71 (1.04–7.08)	0.041		
Bilirubin ≥ 300 μmol/L	2.02 (0.72–5.61)	0.177		
pCLIF-SOFA	1.19 (1.03–1.38)	0.016		
pCLIF-SOFA ≥ 7	2.85 (1.06–7.64)	0.036	5.07 (1.47–17.47)	0.010
PELD	1.05 (1.00–1.13)	0.021		

Odds ratio are expressed as 95% (confidence interval). On multivariable analysis, centre effect was not significant (*p* = 0.13)

*ACLF* acute-on-chronic liver failure, *INR* International Normalized Ratio, *pCLIF-SOFA* Paediatric Chronic Liver Failure-Sequential Organ Failure Assessment score, *PELD* paediatric end-stage liver disease

**Table 4:** Risk factors for mortality (*n* = 33) versus survival (*n* = 97) on day-28

Characteristics	Univariate analysis		Multivariable analysis	
	Odds ratio	*p* value	Odds ratio	*p* value
Growth failure	1.19 (0.48–2.94)	0.69		
ACLF	5.68 (1.57–20.5)	0.008	5.41 (0.75–38.6)	0.092
Sepsis	3.17 (1.34–7.49)	0.009	3.85 (0.82–18.12)	0.087
BA	2.05 (0.87–4.82)	0.098		
INR ≥ 2.5	4.62 (1.88–11.34)	0.001		
Bilirubin ≥ 300 μmol/L	7.34 (2.83–19.0)	0.000		
pCLIF-SOFA	1.52 (1.27–1.81)	0.000	1.76 (1.40–2.20)	0.000
PELD	1.08 (1.03–1.12)	0.000		

Odds ratio are expressed as 95% (confidence interval)

*ACLF* acute-on-chronic liver failure, *BA* biliary atresia, *INR* International Normalized Ratio, *pCLIF-SOFA* Paediatric Chronic Liver Failure-Sequential Organ Failure Assessment score, *PELD* paediatric end-stage liver disease

**Table 5:** Risk factors for composite outcome (*n* = 67) versus survival without liver transplantation (*n* = 63) on day-60

Characteristics	Univariate analysis		Multivariable analysis	
	Odds ratio	*p* value	Odds ratio	*p* value
Comorbidity	0.31 (0.15–0.64)	*0.002*	0.16 (0.05–0.52)	0.003
Growth failure	1.88 (0.84–4.16)	*0.12*		
ACLF	5.8 (1.86–18.32)	*0.002*	2.83 (0.77–11;11)	*0.135*
Sepsis	2.7(1.3–5.62)	*0.005*	2.1 (0.78–5.58)	*0.137*
Biliary atresia	1.5 (0.76–3.06)	*0.23*		
INR ≥ 2.5	3.11 (1.4–6.78)	*0.004*		
Bilirubin ≥ 300 μmol/L	3.5 (1.54–8.05)	*0.003*		
pCLIF-SOFA	1.3 (1.18–1.5)	*0.000*	1.48 (1.25–1.76)	*0.019*
PELD	1.07 (1.03–1.13)	*0.000*		

Odds ratio are expressed as 95% (Confidence Interval). On multivariable analysis, centre effect was not significant (*p* = 0.44)

*ACLF* acute-on-chronic liver failure, *INR* International Normalized Ratio, *pCLIF-SOFA* Paediatric Chronic Liver Failure-Sequential Organ Failure Assessment score, *PELD* paediatric end-stage liver disease

## Discussion

To our knowledge, this is the first study evaluating the characteristics and prognostic factors of a large multicenter cohort of non-transplanted cirrhotic critically ill children admitted to the paediatric intensive care unit. This retrospective study of four large European Paediatric LT centres provided us a comprehensive clinical and biological description of 130 patients admitted over a 5-year period. The main finding is the accuracy of on admission pCLIF-SOFA score for identifying, among survivors, patients transplanted at day-28 and day-60. On multivariable analysis, pCLIF-SOFA was associated with 28-day mortality and composite outcome at day-60.

The high mortality rates observed at day-28 and 60, respectively, of 25.4% and 29.2%, were consistent with published adult data. Adult cohorts of cirrhotic patients admitted to intensive care displayed a mortality ranging from 31 to 40% [14, 15]. In a meta-analysis of 13 adult series, overall intra-hospital mortality at 6 months reaches 75% [1]. Unlike decompensated cirrhosis, ACLF, a syndrome characterized by acute impairment of liver function in response to various kinds of insults in cirrhotic patients, has a very high short-term mortality. Importantly, two definitions of ACLF currently coexist. The Asia Pacific Association for the Study of Liver Diseases (APASL) defined ACLF as an acute liver injury complicating within four weeks of ascites and/or HE [16]. The EASL-CLIF consortium (EASL-CLIF Acute-on-chronic Liver Failure in Cirrhosis, CANONIC) proposed ACLF definition as an impairment of hepatic functions in cirrhotic patients, due to a triggering factor and resulting on at least two organs failure. Considering that the components of SOFA score (liver, kidney, brain, coagulation, circulation and lungs) did not take into account specific characteristics of patients with liver disease, the CANONIC study group adapted it to predict short-term mortality in liver cirrhosis. In adults, ACLF has been proposed to be stratified in three levels. Stage 1 includes (a) patients with single renal impairment (creatinine ≥ 177 μmol/l); (b) patients with single organ failure and creatinine between 133 µmol/l and 168 µmol/l and/or moderate HE, or (c) patients with EH grade 3 or 4 and creatinine between 133 µmol/l and 168 µmol/l. Stage 2 and 3 includes patients with two or three organs failure, respectively. Mortality at day-28 is increasing along with the stage grade to reach 32% and 76% in stage 2 and 3, respectively [11]. Derived and validated from this above mentioned study, the Chronic Liver Failure-Consortium ACLF (CLIF-C ACLF) score is a clinically relevant scoring system that can be used sequentially to stratify the risk of mortality in ACLF patient [18]. Many scores have attempted to predict outcome in patients with decompensated cirrhosis. The CLIF-SOFA score seems to be the most reliable. In a prospective cohort of 62 adult cirrhotic patients admitted in ICU, eight scoring tools were evaluated (including MELD and APACHE II). The CLIF-SOFA had the best accuracy with an AUC of 0.75 (0.62–0.88) confirming larger retrospective cohort of 635 patients and a meta-analysis of 13 studies involving over 2500 patients [1, 14]. In children with advanced cirrhosis, the PELD score is widely used as a reference score for organ allocation in many countries [9, 18]. In contrast to the previous single-centre study suggesting that pCLIF-SOFA had a predictive value for mortality outperforming Child–Pugh and PELD score, our study showed similar performance between pCLIF-SOFA and PELD for 28-day mortality prediction [5]. In addition we showed that a pCLIF-SOFA ≥ 9 had a high accuracy for predicting 28-day mortality.

Salvage liver transplantation in critically ill cirrhotic patients with multiple organ failure demonstrates excellent outcome even though the transplant window is extremely narrow [19]. Data on paediatric ACLF are scarce and, due to heterogeneity in the definitions used in comparison to EASL-CLIF adult criteria, remain challenging. Our study shows that ACLF was present in 18% of the population. It is consistent with the adult’s prevalence of 26% [20], and is close to the only paediatric study single-centre report from India (APASL definition) of 11% rate [21]. In our study, patients presenting ACLF were more likely to die or be transplanted (OR 17.7 95% CI [4.79–65.7], *p* < 0.05). Importantly, no patients who were transplanted died during the 28 and 60 days following PICU admission. The main finding of our study is the identification of a pCLIF-SOFA score ≥ 7 at admission as accurate criteria to identify from all alive patients, those who were transplanted at day-28 and -60.

Beside the pCLIF-SOFA, AKI is an important risk factor for mortality in our study (Additional file 2). Both AKI stage 1 + 2 and 3 are associated with 28-day mortality (data not shown). This association was also observed in a previous study where the mortality reached 53% in patients with AKI associated with ACLF [6]. The pathophysiology is likely to be multifactorial (HRS, hypoperfusion, nephrotoxic drugs) warranting special consideration in the use of nephrotoxic drugs upon admission in order to avoid HRS [22].

In contrast to adult studies that showed strength association between sepsis and mortality in critically ill cirrhotic patients, in our study sepsis was not significantly associated with mortality. In adults, the most severe cases with septic shock, mortality was between 65 to 100% [11, 23]. Data from the prospective CUB-REA study regrouping 32 French adult ICU over a 12-year period, showed that cirrhosis was a risk factors for death in septic shock patients [24]. In our study, SBP due to Gram-negative bacteria was the most common infection. Similarly, another paediatric study showed that SBP, mainly due to *Escherichia coli,* is related to a 39% in-hospital mortality [25]. In adults, SBP is recognized as an independently factors associated to ICU mortality [23]. Liver failure shares many similarities with sepsis with regard to acute inflammation and development of immunoparalysis [26]. Systemic inflammation may be involved in the pathogenesis of ACLF and be a prognostic factor for evolution towards ACLF in patients with acutely decompensated cirrhosis [27, 28]. Although not analysed in detail, presence of comorbidities in our cohort was associated with all outcomes, but 28-day mortality. This goes along with observations in critically ill children where comorbidities are known major prognostic factors in multiple clinical conditions.

Our study has several strengths and limitations. It is the first paediatric collaborative study including four of the largest European paediatric transplant centres. At the same time, the retrospective study design may limit the generalization of the identified prognostic factors although they are consistent with adult data. As such, prospective validation is warranted. Second, inhomogeneous practice among the participating centres due to heterogeneity of both diagnostic and therapeutic approach including transplantation criteria cannot be ruled out. We have attempted to minimize this bias by selecting the largest paediatric European transplant centres, thereby reducing intrinsic variability as shown in previous multicentre paediatric studies involving patients with liver diseases.

## Conclusion

In this first published multicentre retrospective analysis, cirrhotic paediatric patients admitted to PICU are shown to have a severe prognosis. On admission, pCLIF-SOFA is a reliable score for identifying patients transplanted at day-28 and day-60 to those alive without LT and is associated with 28-day mortality and 60-day composite outcome.

## Supplementary information

**Additional file 1.** pCLIF-SOFA and PELD scores. A. Pediatric Chronic Liver Failure Sequential Organ Failure Assessment (p CLIF-SOFA) Score. Values in bold text indicate values defining organ failure. Dopamine, epinephrine and norepinephrine values are in µg/kg/min. *BP *Blood Pressure, *Dopa* Dopamine, *Epi* Epinephrine, *Norepi* Norepinephrine, *ULN* Upper Limit of normal. * In cases arterial blood gas was not performed, non-ventilated patient were scored 0, patient on FiO2=1,0 were scored 4, and mechanically ventilated patient were scored 1. B. Pediatric End-stage Liver Disease (PELD) Score. Growth term: 0.667 when the subject's height or weight is less than 2 S.D. below the mean values for that age. Listing Age Factor term: 0.436 if the subject is under 1 year of age, or if the subject is less than 2 years old AND was listed before the age of 1 year. Serum Bilirubin in mg/dL; Albumin in g/dL.

**Additional file 2.** Kaplan Meier survival curves according to KDIGO stages on day 28.

**Additional file 3. **pCLIF-SOFA, PELD, PIM2 scores for primary and secondary outcomes.

## Data Availability

The dataset used and/or analysed during the current study are available from the corresponding author on reasonable request.

## Abbreviations

ACLF: Acute-on-chronic liver failure
AKI: Acute kidney injury
CLD: Chronic liver disease
EASL-CLIF: European Association for the Study of Liver-Chronic Liver Failure
HE: Hepatic encephalopathy
KDIGO: Kidney Disease Improving Global Outcomes
LT: Liver transplantation
pCLIF-SOFA: Paediatric Chronic Liver Failure-Sequential Organ Failure Assessment Score
PELD: Paediatric end-stage liver disease
PICU: Paediatric intensive care units
PIM2: Paediatric index of mortality
