# Supplementary figures and images for: pCLIF-SOFA is a reliable outcome prognostication score of critically ill children with cirrhosis: an ESPNIC multicentre study
Source: Ann Intensive Care. 2020 Oct 14;10:137. doi: 10.1186/s13613-020-00753-w (PMC7560665; doi:10.1186/s13613-020-00753-w)

## Slide 1
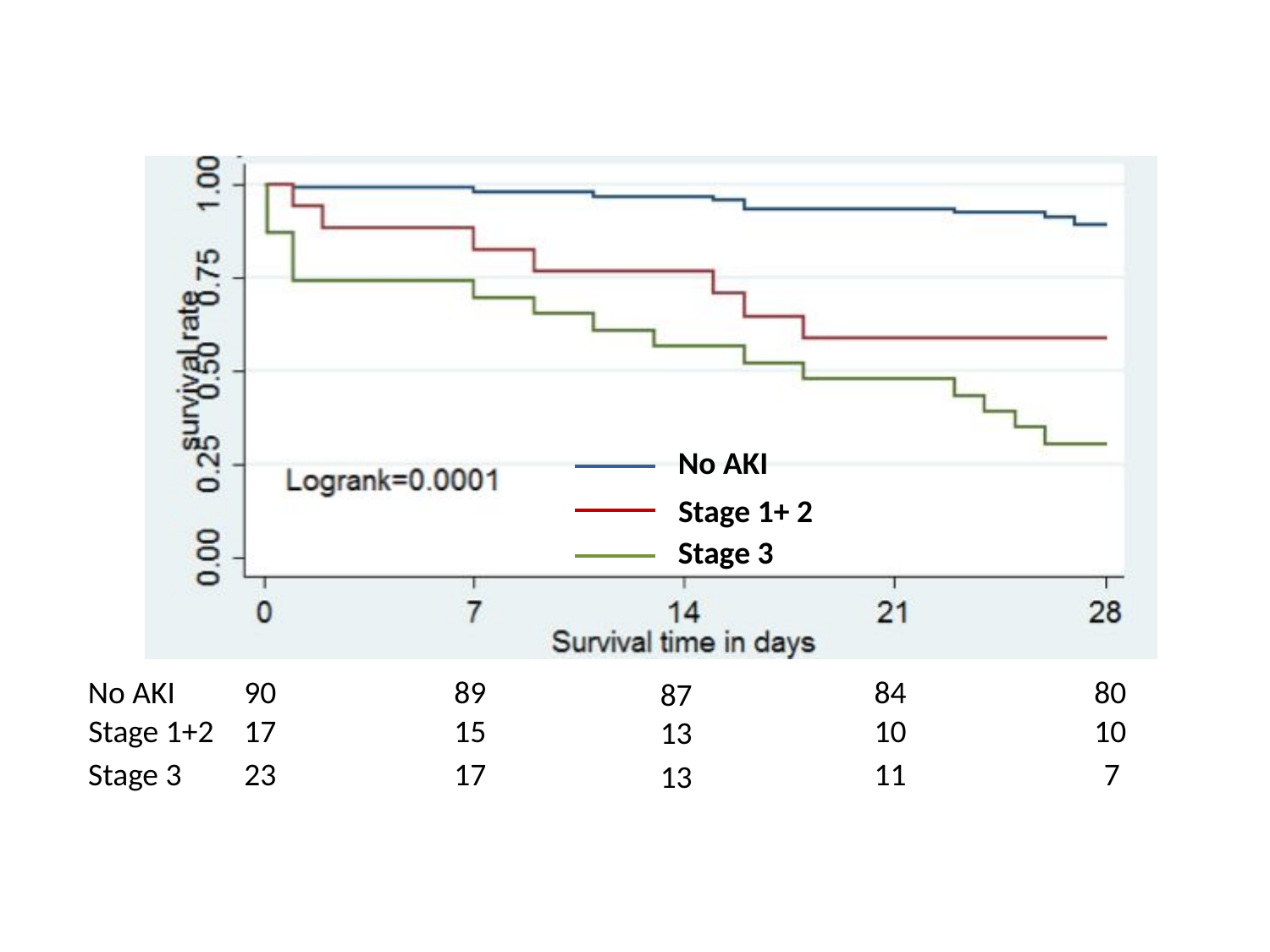

No AKI
Stage 1+ 2
Stage 3
No AKI
90
89
84
80
87
Stage 1+2
17
15
10
10
13
Stage 3
23
17
11
7
13

Supplement: Supplementary file 1 — Additional file 1. pCLIF-SOFA and PELD scores. A. Pediatric Chronic Liver Failure Sequential Organ Failure Assessment (p CLIF-SOFA) Score. Values in bold text indicate values defining organ failure. Dopamine, epinephrine and norepinephrine values are in µg/kg/min. BP Blood Pressure, Dopa Dopamine, Epi Epinephrine, Norepi Norepinephrine, ULN Upper Limit of normal. * In cases arterial blood gas was not performed, non-ventilated patient were scored 0, patient on FiO2=1,0 were scored 4, and mechanically ventilated patient were scored 1. B. Pediatric End-stage Liver Disease (PELD) Score. Growth term: 0.667 when the subject's height or weight is less than 2 S.D. below the mean values for that age. Listing Age Factor term: 0.436 if the subject is under 1 year of age, or if the subject is less than 2 years old AND was listed before the age of 1 year. Serum Bilirubin in mg/dL; Albumin in g/dL. [file 13613_2020_753_MOESM1_ESM.pptx]
