# Supplementary material for: pCLIF-SOFA is a reliable outcome prognostication score of critically ill children with cirrhosis: an ESPNIC multicentre study
Source: Ann Intensive Care. 2020 Oct 14;10:137. doi: 10.1186/s13613-020-00753-w (PMC7560665; doi:10.1186/s13613-020-00753-w)
Supplement: Supplementary file 2 — Additional file 2. Kaplan Meier survival curves according to KDIGO stages on day 28. [file 13613_2020_753_MOESM2_ESM.pptx]

## Slide 1
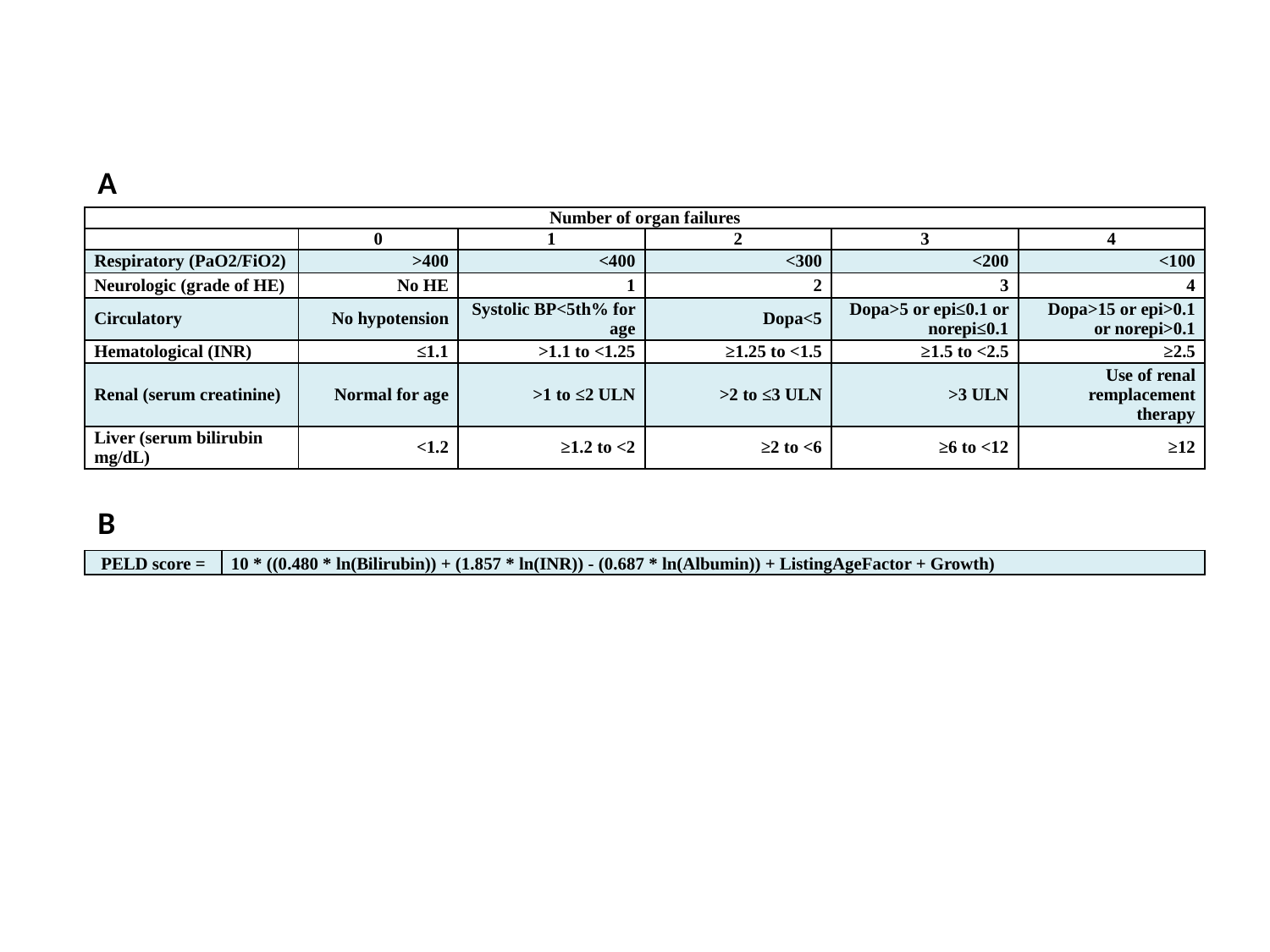

A
| Number of organ failures | | | | | |
| --- | --- | --- | --- | --- | --- |
| | 0 | 1 | 2 | 3 | 4 |
| Respiratory (PaO2/FiO2) | >400 | <400 | <300 | <200 | <100 |
| Neurologic (grade of HE) | No HE | 1 | 2 | 3 | 4 |
| Circulatory | No hypotension | Systolic BP<5th% for age | Dopa<5 | Dopa>5 or epi≤0.1 or norepi≤0.1 | Dopa>15 or epi>0.1 or norepi>0.1 |
| Hematological (INR) | ≤1.1 | >1.1 to <1.25 | ≥1.25 to <1.5 | ≥1.5 to <2.5 | ≥2.5 |
| Renal (serum creatinine) | Normal for age | >1 to ≤2 ULN | >2 to ≤3 ULN | >3 ULN | Use of renal remplacement therapy |
| Liver (serum bilirubin mg/dL) | <1.2 | ≥1.2 to <2 | ≥2 to <6 | ≥6 to <12 | ≥12 |
*
B
| PELD score = | 10 \* ((0.480 \* ln(Bilirubin)) + (1.857 \* ln(INR)) - (0.687 \* ln(Albumin)) + ListingAgeFactor + Growth) |
| --- | --- |
