# Supplementary material for: pCLIF-SOFA is a reliable outcome prognostication score of critically ill children with cirrhosis: an ESPNIC multicentre study
Source: Ann Intensive Care. 2020 Oct 14;10:137. doi: 10.1186/s13613-020-00753-w (PMC7560665; doi:10.1186/s13613-020-00753-w)
Supplement: Supplementary file 3 — Additional file 3. pCLIF-SOFA, PELD, PIM2 scores for primary and secondary outcomes. [file 13613_2020_753_MOESM3_ESM.doc]

Additional file 3.

pCLIF-SOFA, PELD, PIM2 scores

| **Primary outcome: Liver transplantation at day-28 in survivors** | | | |
| --- | --- | --- | --- |
|  | Group 1  (n=22) | Group 2  (n=75) | *p value* |
| pCLIF-SOFA | 9.2±3.8 | 7.1±3.0 | 0.0076 |
| PELD | 22.3±9.6 | 13.4±13.2 | 0.009 |
| PIM2* | 13.0% | 14.5% | 0.66 |
| **Secondary outcome 1: Liver transplantation at day-60 in survivors** | | | |
|  | Group 4  (n=29) | Group 5  (n=63) | *p value* |
| pCLIF-SOFA | 8.7±3.5 | 6.8±3.0 | 0.011 |
| PELD | 20.0±10.5 | 12.6±13.3 | 0.021 |
| PIM2* | 13.8% | 14.7% | 0.78 |
| **Secondary outcome 2: Mortality on day-28 without Liver transplantation (n=108)** | | | |
|  | Group 3  (n=33) | Group 2  (n=75) | *p value* |
| pCLIF-SOFA | 11.9±3.8 | 7.1±3.0 | <0.0001 |
| PELD | 25.4±11.0 | 13.4±13.2 | <0.0001 |
| PIM2* | 13.8% | 14.5% | 0.805 |
| **Secondary outcome 3: combined criterion including Liver transplantation OR death at day-60 (n=130)** | | | |
|  | Group 4 +6  (n=67) | Group 5  (n=63) | *p value* |
| pCLIF-SOFA | 10.4±3.9 | 6.8±3.0 | <0.0001 |
| PELD | 22.9±11.1 | 12.6±13.4 | <0.0001 |
| PIM2* | 13.4% | 14.7% | <0.0001 |

Data are expressed as mean±SD; * PIM2 predicted mortality is expressed in %
